# Cobalt, titanium and PMMA bone cement debris influence on mouse osteoblast cell elasticity, spring constant and calcium production activity

**DOI:** 10.1039/c5ra15390e

**Published:** 2015-10-02

**Authors:** Emily Callard Preedy, Stefano Perni, Polina Prokopovich

**Affiliations:** a School of Pharmacy and Pharmaceutical Sciences, Cardiff University, Cardiff, UK. Email: prokopovichp@cardiff.ac.uk; Fax: +44 (0)29 208 74149; Tel: +44 (0)29 208 75820; b Department of Biological Engineering, Massachusetts Institute of Technology, Cambridge, USA

## Abstract

Periprosthetic osteolysis and implant loosening are the outcomes of wear debris generation in total joint replacements. Wear debris formed from the implanted materials consisting of metals, polymers, ceramic and bone cement initiate the immune system response. Often osteoblasts, the principal cell type in bone tissue adjacent to the prostheses, are directly impacted. In this study, the influence of cobalt, titanium and PMMA bone cement particles of different sizes, charges and compositions on mouse osteoblast adhesion, nanomechanics (elasticity and spring constant) and metabolic activity were investigated. These studies were accompanied by osteoblast mineralisation experiments and cell uptake after exposure to particles at defined time points. Our results demonstrate that alteration of the nanomechanical properties are mainly dependent on the metal type rather than nanoparticles size and concentration. Moreover, despite uptake increasing over exposure time, the cell characteristics exhibit changes predominately after the first 24 hours, highlighting that the cell responses to nanoparticle exposure are not cumulative. Understanding these processes is critical to expanding our knowledge of implant loosening and elucidating the nature of prosthetic joint failure.

## Abstract 2

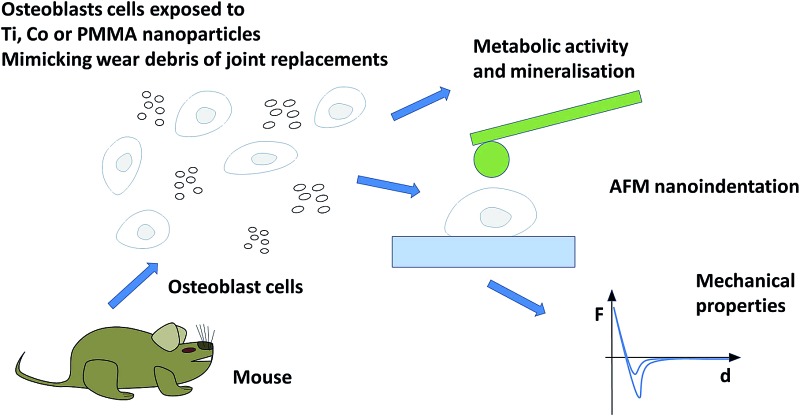
Osteoblast cells exhibit nanomechanical changes of after exposure to titanium, cobalt and PMMA particles simulating joint devices replacements wear debris.

## 1 Introduction

Other than the onset of an infection, deterioration of the material components of the implanted biomedical device is a major complication in total joint arthroplasty (TJA), which coincides with a biological response to the particles released from the damaged device. As people are generally living longer, then it is necessary for biomedical implant longevity to also rise with the aging population. Yet, aseptic loosening has been reported to mature within 15–20 years post total joint replacement surgery,^1^ and is inevitable.

Aseptic loosening is debilitating to patients^2^ and results from the subtle wear of the articulating surfaces from every day movements experienced during simple activities such as walking. The major implication of the slow destruction of the materials is that signs and symptoms of the damage do not present clinically until late stages of implant failure. Furthermore, the biological response, or the destruction of the periprosthetic tissue *i.e.* osteolysis, is the result of an inflammatory response due to the defence mechanisms of the immune system responding to the wear debris.^3,4^ Initially, the foreign particles are phagocytosed by macrophages which governs the release of proinflammatory cytokines;^5^ as the proinflammatory cascade continues the cellular mediators, cytokines, activate osteoclasts, precursor cells of hemopoietic lineage leading to differentiation, and maturation.^5–9^ In turn these defence cells inhibit and suppress differentiation and proliferation of osteoprogenitor cells, and therefore the function of osteoblast cells, even causing apoptosis.^5^

These biochemical interactions have been widely investigated^2,10–12^ and found dependent on the type, size, and number of wear particles.^13^ Simultaneous to these biological responses, cells may also undergo mechanotransduction and adhesive changes.^14,15^ These changes will naturally take place within cells as they are living entities that possess structural, as well as physical properties; these are characteristics that aid cells survival enabling them to withstand physiological changes, such as mechanical stimuli that may occur both internally and externally.^16^ Any disruptions to the usual characteristics of the cell will potentially upset its integrity and biological function.^14,16^ For example, osteoprogenitor cells can sense the rigidity of their surroundings regulating their shape, internal cytoskeletal tension, stiffness and thereby proliferation.^14^ Yet these physical characteristics have been sidelined to biological responses for many years, and only recently have been thought as contributing factors of pathologies, with the notion that mechanical signals eventually are converted into biological and chemical stimuli governing the growth, differentiation, migration, and apoptosis of cells.^16^

With enhanced microscopy techniques such as the atomic force microscope (AFM), detailed quantitative analysis of the nanomechanical and adhesive characteristics can now been investigated.^14,15^ As cells do not only respond to external forces but internal ones too, some responses may even overcome chemical stimuli which in turn could have similar outcomes to the function, proliferation, differentiation and apoptosis of cells.^14,15,17^ The function and integrity of cells is governed by their cytoskeleton which is responsible for their structural and mechanical properties.^15,18,19^ For instance, following a TJA bone remodelling cells also adapt not only to the biological changes but to an altered mechanical environment,^20^ which is the direct effect of the redistribution of load, and inherently stress, especially when the femoral head is replaced in a hip replacement.^20^

Bone cells such as osteoblasts are essential in bone remodelling and osseointegration of biomedical implants.^21–23^ Adhesive properties of cells are therefore required to establish successful integration, as well as the biocompatibility of the implanted device material which is essentially the substrate for these osteoblast cells.^21^ Cells tend to adhere and spread better to rigid substrates^15^ but spreading also increases cortical stiffness.^15,24^ Internal tension control allows cells to finely tune their own stiffness and is determined by the actin cytoskeleton.^24^ It is therefore important to gain further understanding in the mechanics and mechanotransduction characteristics^24^ and how wear debris influence such properties.

This study aims to premier mouse osteoblasts adhesive forces, nanomechanical properties and metabolic activity post exposure to metal and bone cement particles simulating wear debris generated by joint replacement devices of various sizes, charges, compositions and concentrations in order to gain insight on the biological responses of bone cells to stimuli that could lead to implant failure.

## 2 Methods and materials

### 2.1. Particles

Commercially available nanoparticles were obtained of various sizes and compositions from Sigma-Aldrich, UK. For cobalt (Co) nanoparticles (NPs), two samples were employed:

• Co elemental, 30 nm diameter (referred as ‘Co 30 nm’ throughout the text).

• Co(ii,iii) oxide, 50 nm diameter (referred as ‘Co 50 nm’ throughout the text).

Three samples of titanium (Ti) were employed:

• Ti elemental, 30 nm diameter.

• Ti(iv) oxide anatase, 25 nm diameter.

• Mixture of Ti(iv) oxide rutile and anatase, 100 nm diameter.

They are referred to as ‘Ti 30 nm’, ‘Ti 25 nm’ and ‘Ti 100 nm’, respectively, throughout the text.

Polymethyl-methacrylate (PMMA) bone cement particles were generated using a single station pin on plate in-house built wear simulator under constant applied load in lubricated conditions as detailed.^25^ PMMA pins for the wear process were prepared from PMMA bone cement obtained by manually mixing the powder phase consisting of poly-methyl-methacrylate (4.1 g), barium sulphate (0.46 g) and benzoyl peroxide (0.1 g) with the liquid phase consisting of methyl-methacrylate (1.96 g) and *N-N* dimethyl-*p*-toluidine (0.04 g) under constant stirring until the powder was fully wetted.^26,27^ The mixture was subsequently inserted into the mould at an approximate dough time of 1 minute. The filled mould was pressed between two glass plates for 1 hour. After the cement had hardened, it was pulled out from the mould and stored under dark, sterile conditions at room temperature. Prior to wear experimentation bone cement specimens were conditioned at 37 °C for 24 hours.

### 2.2. Cell culture

Murine MC3T3-E1 osteoblasts cells (Sigma, UK) were routinely cultured in α-MEM (Life Technologies), supplemented with 10% (v/v) FBS, 1% (v/v) of solution penicillin (5000 U ml^–1^) and streptomycin (5 mg ml^–1^) (Gibco Invitrogen). Trypsin (Gibco Invitrogen) was used when cells were about 70% confluent in order to passage and count. The cells were incubated at 37 °C in a humidified atmosphere containing 5% CO_2_.

Cells were seeded in 24-well plates at a density of 6000 cells per well and cultured for 24 hours on sterilised polystyrene slides placed inside the well. For each type of particles a stock solution of the particles suspended in culture media was prepared at 5 mg ml^–1^ and the appropriate amount was added to each well to reach final concentrations of 5, 12.5, 25 and 50 μg ml^–1^ for Co and Ti nanoparticles and 5, 25, 50, 100 and 200 μg ml^–1^ for PMMA bone cement particles; cells were then incubated with the nanoparticles for 24 h, 48 h and 72 h. Control samples consisting of cells not exposed to particles and cultured in the same conditions were used for comparison with treated cells.

### 2.3. Metabolic activity assay

MTT (3-(4,5-dimethythiazol-2yl)-2,5-diphenyltetrazolium bromide) assay was used to determine the effects of the metal and PMMA bone cement particles on mouse osteoblast (MC3T3) viability. It is a colorimetric assay and depends on the metabolic activity of the cell as rapidly dividing cells exhibit high rates of MTT reduction. Cells were initially cultured and exposed to particles as stated above; after the chosen exposure time, the media was replaced with phenol red-free medium and 80 μl of MTT stock solution (5 mg ml^–1^) was added to each well and incubated at 37 °C in humidified atmosphere containing 5% CO_2_ for 2 hour. The metabolised MTT, formazan, was re-suspended with 800 μl of dimethylsulfoxide (DMSO). 200 μl were transferred to a 96-well plate and absorbance at 560 nm was read using a spectrophotometer (ELISA Reader Labtech LT-5000MS). All experiments were performed in triplicates.

### 2.4. Osteoblast mineralisation

Mouse osteoblast (MC3T3-E1) cells were grown with metal (Co or Ti) nanoparticles or PMMA bone cement particles for the period of 21 days. Particles and medium were changed every 3rd or 4th day. On day 21 all of the medium was removed from the wells and replaced with 100 μl of glutaraldehyde (Sigma-Aldrich, UK) 10% (v/v) in PBS followed by incubation for 10 min. The glutaraldehyde was then removed and each well was washed three times using 100 μl of PBS. 100 μl of alizarin red staining (ARS) (Sigma-Aldrich, UK) 1% (w/v) was pipetted into each well and incubated for 20 min. The ARS was completely removed and each well was washed with Milli-Q water. 100 μl of acetic acid 10% (v/v) was added to each well and incubated for 30 min. 50 μl samples from each well were taken and transferred into a fresh 96 well plate. The absorbance at 405 nm was measured with Labtech-LT5000MS ELISA.^28^

All experiments were performed in triplicates.

### 2.5. Zeta potential and size of particles measurements

Particles size was measured through dynamic light scattering (DLS), zeta potential of nanoparticles was measured using laser doppler micro-electrophoresis which calculates the electrophoretic mobility to evaluate the zeta potential. Both characterisations were carried out using Malvern Zetasizer Nano ZS Nano series (Malvern, UK). All measurements were performed on Ti or Co nanoparticles or PMMA bone cement particles suspensions at 5 μg ml^–1^ prepared from a stock solution of 5 mg ml^–1^.

PMMA debris were imaged after gold coating for 15 seconds using a sputter coater from Agar (Model 109A, Stansted, Essex, UK), with a mixture of gold and palladium (80% and 20%, respectively) in argon. Once coated, SEM (XB1540, Carl Zeiss, Germany) was used to obtain images. Size determination was performed using ImageJ software determining the diameter of a circle with the same projected area of the particles.

### 2.6. Cell nanomechanical properties measurements

All AFM force measurements were conducted in an open liquid cell as described in,^29^ using PBS as the aqueous phase. A triangular tipless cantilevers (Bruker, UK) with a nominal spring constants (*K*_cantilever_) of 0.1 N m^–1^ was used; the actual spring constant of the AFM cantilever was determined using the Sader method.^30,31^ Borosilicate glass beads (10 μm in diameter) were glued onto the cantilever and served as cell indentor. In order to prevent indentations depth greater than 400–500 nm, the maximum applied load was set, after preliminary tests, to 1 nN or 2 nN depending on the samples. At least 15 cells were analysed for each sample, at each concentration of particles and at each time point (24, 48, and 72 hours). Cells were first located and then at least 20 approaching and retracting *z*-piezo coordinates *vs.* deflection curves were extracted from randomly selected points on the surface of each cell avoiding the peri-nuclear region. Experiments were performed in triplicates.

#### 2.6.1. Cell elasticity and spring constant determination

The approaching part (trace) of the AFM curves was used to calculate the nanomechanical properties of the cells. The Young modulus of the cell surface location under investigation was determined fitting the Hertz model (eqn (1)) to the first part of the indentation *vs.* force curve after contact between AFM tip and cell surface.1
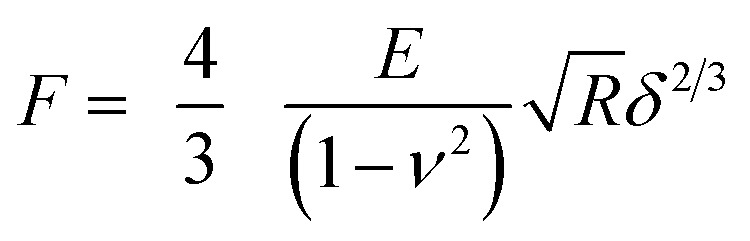
where: *F* = force recorded by AFM, *E* = Young modulus, *R* = radius of the spherical indentor (5 μm), *ν* = Poisson ratio (assumed 0.5), *δ* = indentation depth.

The spring constant of the cell surface in the location probed was determined through the slope of the curve after the Hertzian regime according to:

2 *F* = *K*_b_*δ*

where: *F* = force recorded by AFM, *K*_b_ = spring constant of the cell, *δ* = indentation depth.

Both models require the determination of the separation between cell surface and AFM tip (*δ*), this was calculated from the coordinates (*z*-piezo) of the trace curve assuming that the point of contact corresponded to the local minimum of force; from this:

3 *δ* = |*z* – *z*_0_| – *d*_cant_

where: *z*_0_ = *z*-piezo value of the minimum of the trace curve, *z* = *z*-piezo value of the trace curve, *d*_cant_ = cantilever deflection, *δ* = indentation depth and

4 *F* = *K*_Cantilever_*d*_cant_

Both eqn (1) and (2) were fitted to the data using the least squares method through an in-house written FORTRAN code.

Overall surface heterogeneity of nanomechanical properties was studied through the spatial distribution of *E* and *K*_b_.

#### 2.6.2. Cell adhesion force

The adhesion forces between a cell and AFM tip were determined as the minimum value of the retracting (retrace) part of the AFM curve.

### 2.7. Cell metal (Co or Ti) and Ba uptake quantification

Quantification of the cells uptake of the metal (Co and Ti) nanoparticles and PMMA bone cement (through Ba determination) particles was achieved by using inductively coupled plasma-mass spectroscopy (ICP-MS). The cells were grown and exposed to the particles according to procedure described. At the chosen period, all media was removed from each well, cells were washed twice with sterile PBS, and 500 μl of sub-boiled nitric acid (1 : 1) was added to each well. The 24-well plate was then placed in an incubator for 24 hours at 60 °C in order to digest the cells. After 24 hours in the incubator, from each well 400 μl of the solution was transferred into a 15 ml polypropylene tube and filled to a total volume of 8 ml with Milli-Q water. ICP-MS analysis was carried out at sample rate of 1.5 ml min^–1^ and at characteristic wavelengths of 288.616 nm, 334.940 nm and 233.527 nm for cobalt, titanium and barium ion determination, respectively, on Optima 2100DV OES (Perkin-Elmer, Waltham, MA, USA) against the Primar 28 element standard.

All experiments were performed independently at least 3 times, and each experiment comprised 3 parallel samples. Results are given as mean ± standard deviation.

### 2.8. Statistical analysis

Comparison of the effect of Ti, Co and PMMA bone cement particles on mechanical properties of mouse osteoblast (MC3T3-E1) cells was performed through ANOVA test followed *post hoc* by Tukey's test for individual pairs of data sets (*p* < 0.05). Adhesion forces were compared using the Kruskal–Wallis test followed *post hoc* with a Dunn's test for individual pairs of data sets. Statistical analysis was performed using SPSS.

## 3 Results

### 3.1. Size and charge of particles

**Fig. 1:**
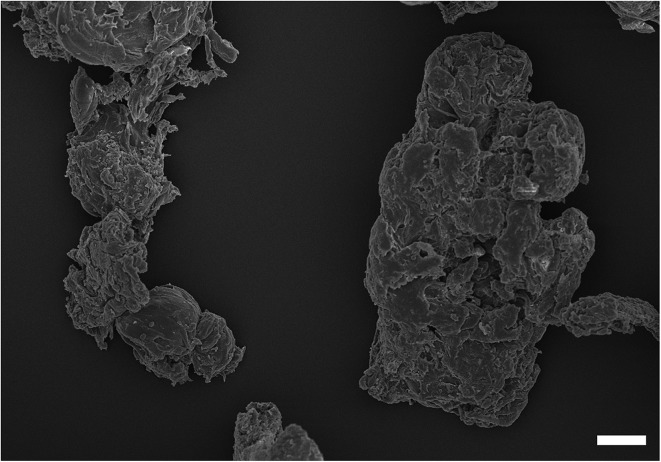
Examples of SEM image of PMMA wear debris. Bar correspond to 1 microns.

The zeta potentials of all nanoparticles and cells are given Table 1. All particles, all metals and PMMA, had negative zeta potentials. Both compositions of cobalt nanoparticles had negative potentials of around –20 mV, whilst 30 nm titanium nanoparticles displayed the lowest overall negative charge at –44 mV. The smallest sized (25 nm) and largest titanium nanoparticles (100 nm) had similar values at around –27 mV. PMMA particles also had a negative potential of –12 mV. An example of PMMA wear particles image is shown in Fig. 1, the shape did not appear spherical and the equivalent diameter was about 5 microns.

**Table 1:** Zeta potential of particles employed and pH of MC3T3-E1 media solution containing them

Particles	pH	Zeta potential (mV)	Size (nm)
Co 30 nm	8.33	–19.4 ± 1.0	27.3 ± 2.1
CoO_2_ 50 nm	7.19	–20.4 ± 0.8	49.3 ± 0.6
Ti 30 nm	7.07	–44.7 ± 1.9	27.7 ± 3.5
TiO_2_ 100 nm	7.15	–28.9 ± 1.7	99.0 ± 2.6
TiO_2_ 25 nm	7.16	–26.8 ± 0.5	27.7 ± 2.3
PMMA bone cement	7.17	–12.1 ± 0.8	5.2 ± 1.8 × 10^3^

### 3.2. Metabolic activity

**Fig. 2:**
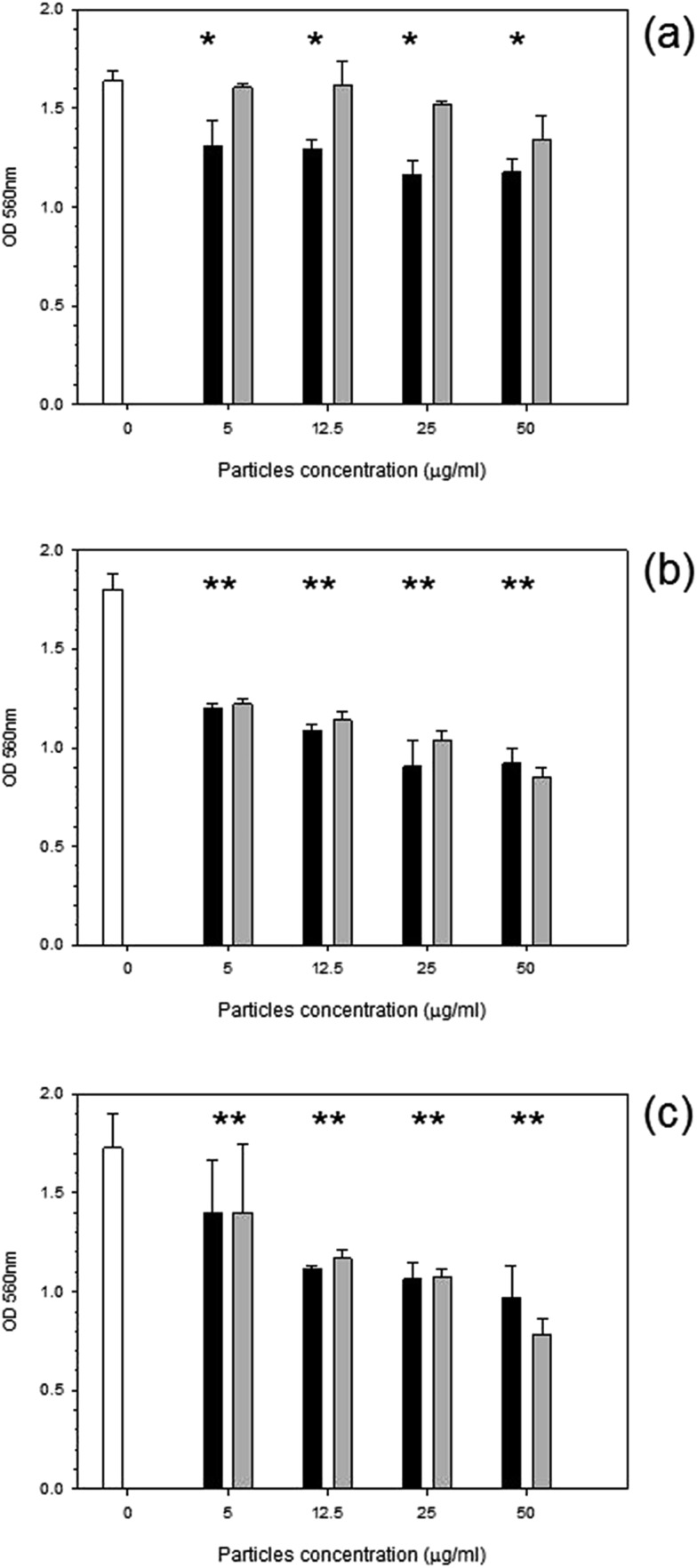
MTT results of MC3T3-E1 cells exposed to cobalt nanoparticles for (a) 24 h, (b) 48 h and (c) 72 h. 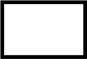 Control, 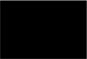 Co 30 nm and 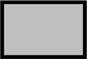 Co 50 nm. *Represent samples statistically different from samples not exposed to particles.

MTT was used to assess the metabolic activity of mouse osteoblasts. The metabolic activity gradually declined with time over a 72 hours period with cobalt nanoparticles compared to control samples (Fig. 2). Results for the control sample over the 3 days remained consistent at around 1.8 OD; for Co 30 nm, there is a deep decline in metabolic activity with the lowest concentration of only 5 μg ml^–1^ for all time points (*p* < 0.05), reducing to around 1.3 OD, for Co 50 nm at 24 hours the decrease in metabolic activity was not as pronounced as for Co 30 nm and was not different from control samples (*p* > 0.05); however, after 48 hours and 72 hours metabolic activity for Co 50 nm resembled that of Co 30 nm (*p* > 0.05), thus lower than control samples (*p* < 0.05). No effect of concentration was noticed for both types of Co nanoparticles (*p* > 0.05).

**Fig. 3:**
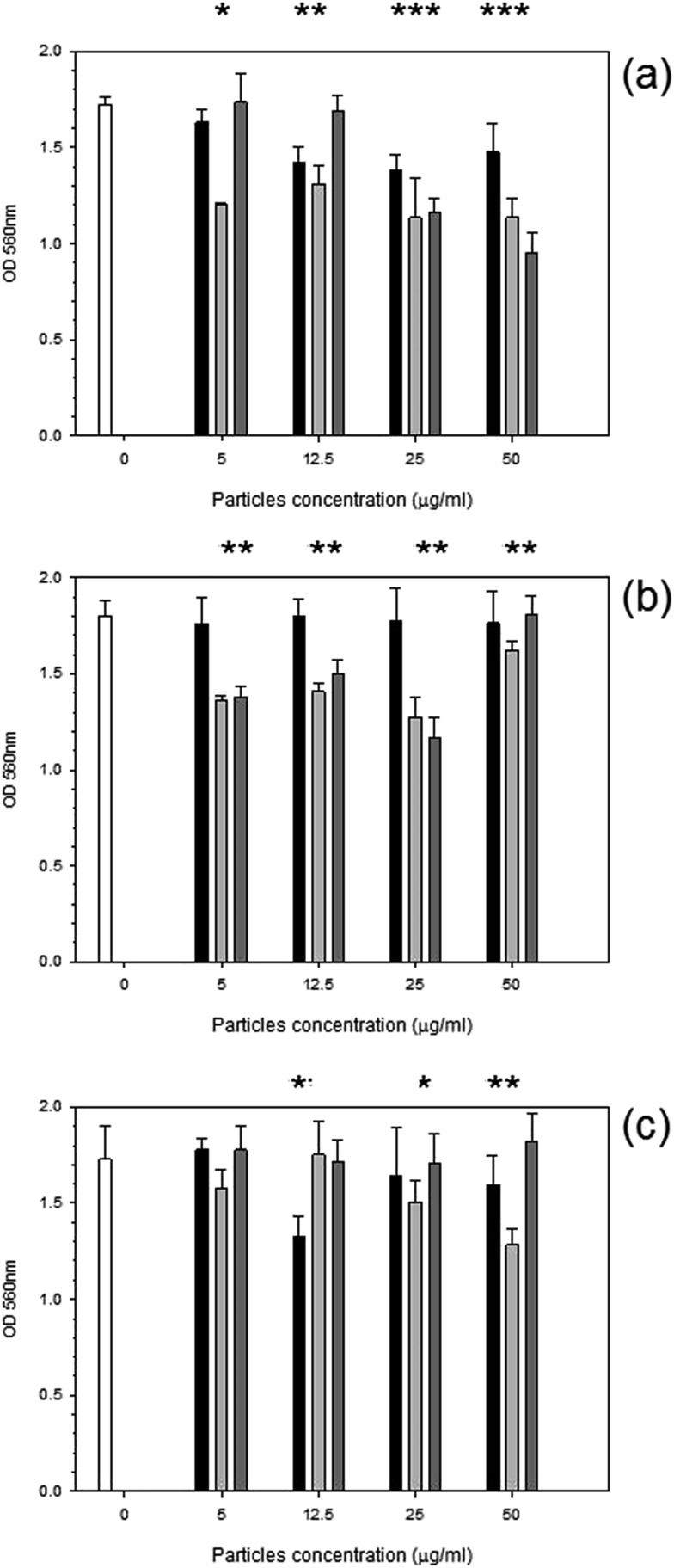
MTT results of MC3T3-E1 cells exposed to titanium nanoparticles for (a) 24 h, (b) 48 h and (c) 72 h. 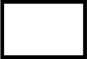 Control, 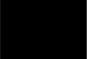 Ti 30 nm, 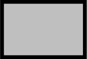 Ti 25 nm and 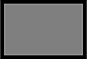 Ti 100 nm. *Represent samples statistically different from samples not exposed to particles.

Similarly with titanium nanoparticles exposure (Fig. 3); after 24 hours a general decline in metabolic activity was observed with Ti 30 nm nanoparticles (*p* < 0.05) without effect of concentration (*p* > 0.05). Initially after 24 hours the smallest titanium particle of 25 nm caused a decrease in metabolic activity to 1.2 OD with concentration of particles at >5 μg ml^–1^ (*p* < 0.05). Ti 100 nm demonstrated a reduction compared to control at concentrations of 25 and 50 μg ml^–1^ after 24 hours (*p* < 0.05). No effect was observed after 48 hours for Ti 30 nm with a similar metabolic activity to the control (*p* > 0.05); yet both Ti 25 nm and Ti 100 nm reduced the metabolic activity to 1.4 OD regardless of the concentration (*p* > 0.05). After 72 hours the same pattern was demonstrated *i.e.* a slight reduction was observed from the control cells for all Ti nanoparticles concentrations.

**Fig. 4:**
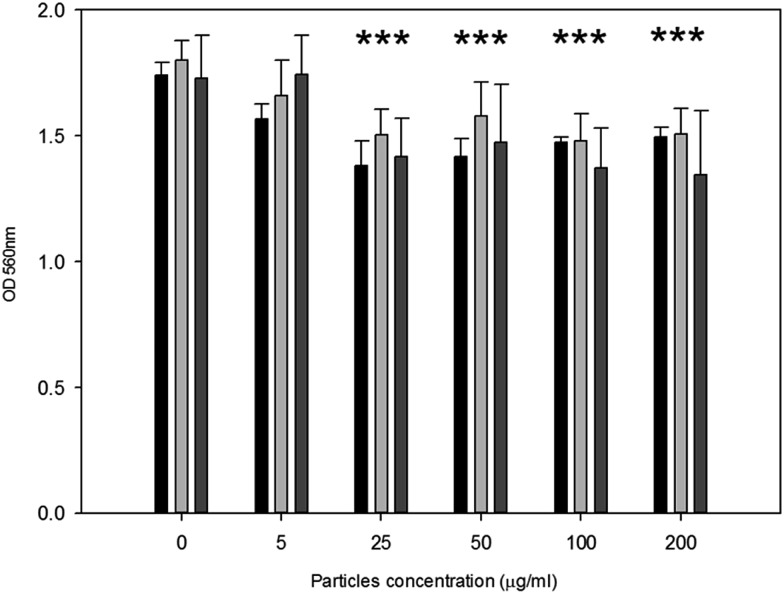
MTT results of MC3T3-E1 cells exposed to PMMA particles for (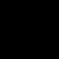) 24 h, (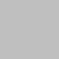) 48 h and (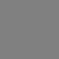) 72 h. *Represent samples statistically different from samples not exposed to particles.

Metabolic activity after exposure to PMMA particles is shown in Fig. 4. A general decrease in metabolic activity is observed with concentration of particles above 25 μg ml^–1^ compared to control samples after each of the three exposure time (*p* < 0.05).

### 3.3. Osteoblast mineralisation ability

**Fig. 5:**
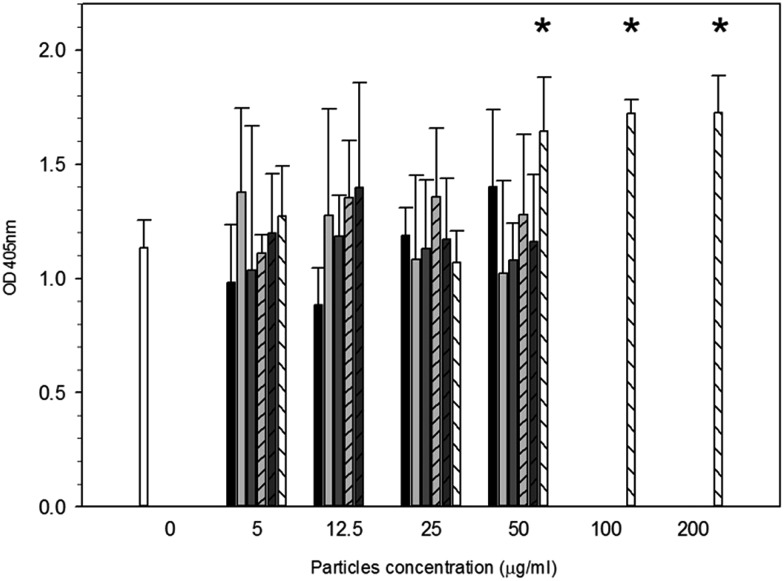
Osteoblast mineralisation ability after exposure to particles. 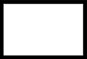 Control, 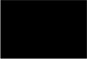 Ti 30 nm, 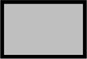 Ti 25 nm, 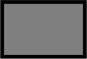 Ti 100 nm, 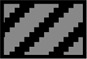 Co 25 nm, 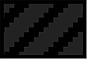 Co 50 nm and 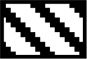 PMMA. *Represent samples statistically different from samples not exposed to particles.

Alizarin red assay was used to determine the production of calcium after 21 days of particle exposure (Fig. 5). After exposure to any of the titanium or cobalt nanoparticles tested for 21 days, no significant difference in the calcium produced by osteoblast was detected compared to samples incubated without nanoparticles (*p* > 0.05) regardless of the concentration employed. For PMMA wear debris, no difference compared to control was determined in calcium production for concentrations up to 25 μg ml^–1^ (*p* > 0.05), above this concentration an increase was recorded (*p* < 0.05) and statistical difference was found among the concentrations tested above 25 μg ml^–1^ (*p* > 0.05).

### 3.4. Nanomechanical properties

**Fig. 6:**
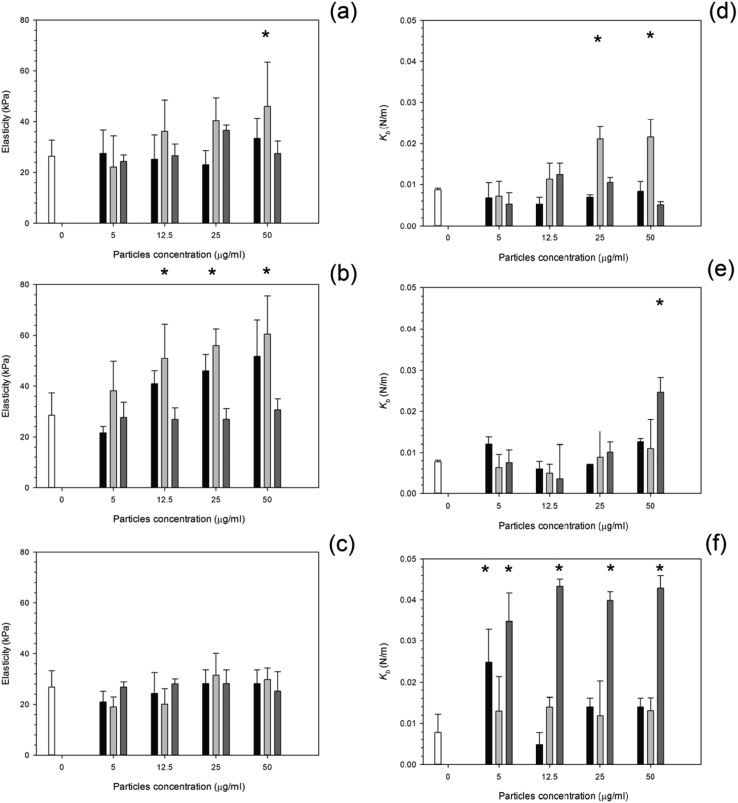
Mean cell elasticity and spring constant of MC3T3-E1 cells exposed to titanium nanoparticles for (a and d) 24 h, (b and e) 48 h and (c and f) 72 h. 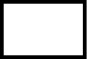 Control, 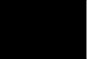 Ti 30 nm, 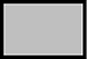 Ti 25 nm and 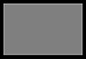 Ti 100 nm. *Represent samples statistically different from samples not exposed to particles.

After 24 hours, cells exposed to titanium (Fig. 6) had the same elasticity as control samples *i.e.* cells not exposed to any particles (*p* > 0.05) apart for the highest concentration of Ti 25 nm. The elasticity of control cells remained constant over the 3 day period with an elastic value of around 28 kPa (*p* > 0.05). Over time, at 48 hours, an increase in elasticity is clearly demonstrated for Ti 25 nm, however no change was seen with the largest particles of 100 nm and for Ti elemental (30 nm particles) (*p* > 0.05). For Ti 25 nm a general increase with increasing concentration is observed from 5 μg ml^–1^ to 12.5 μg ml^–1^ (*p* < 0.05), after this no effect of nanoparticles concentration was detected (*p* > 0.05). After 72 hours of exposure no effect on cell elasticity was detected for any nanoparticles at any concentration (*p* > 0.05).

**Fig. 7:**
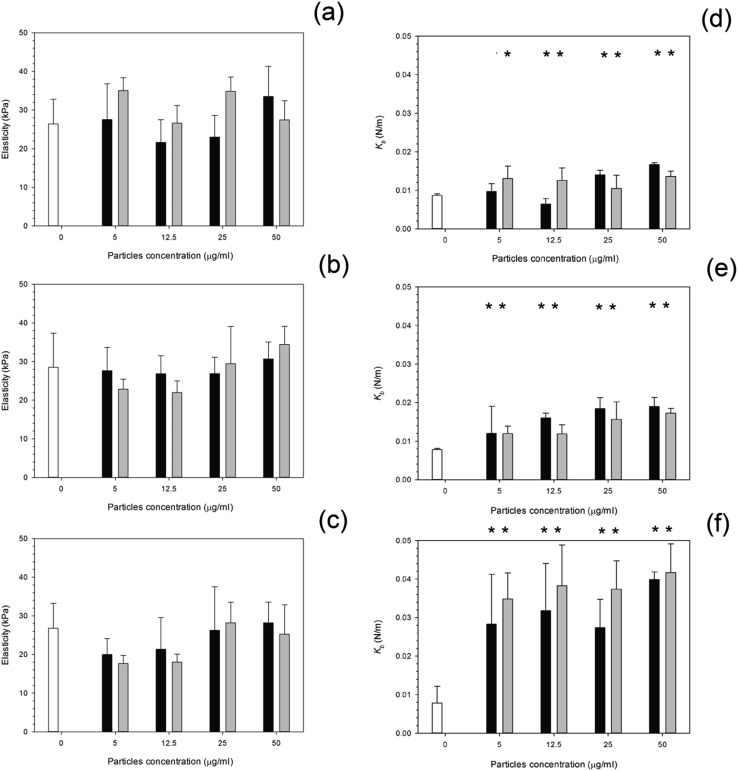
Mean cell elasticity and spring constant of MC3T3-E1 cells exposed to cobalt nanoparticles for (a and d) 24 h, (b and e) 48 h and (c and f) 72 h. 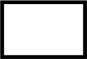 Control, 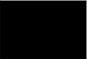 Co 30 nm and 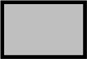 Co 50 nm. *Represent samples statistically different from samples not exposed to particles.

Elasticity results for cobalt nanoparticles are given in Fig. 7. Over all three time points: 24, 48, and 72 hours, the elasticity of osteoblast cells did not change compared to control samples regardless of the nanoparticles type and concentration (*p* > 0.05).

**Fig. 8:**
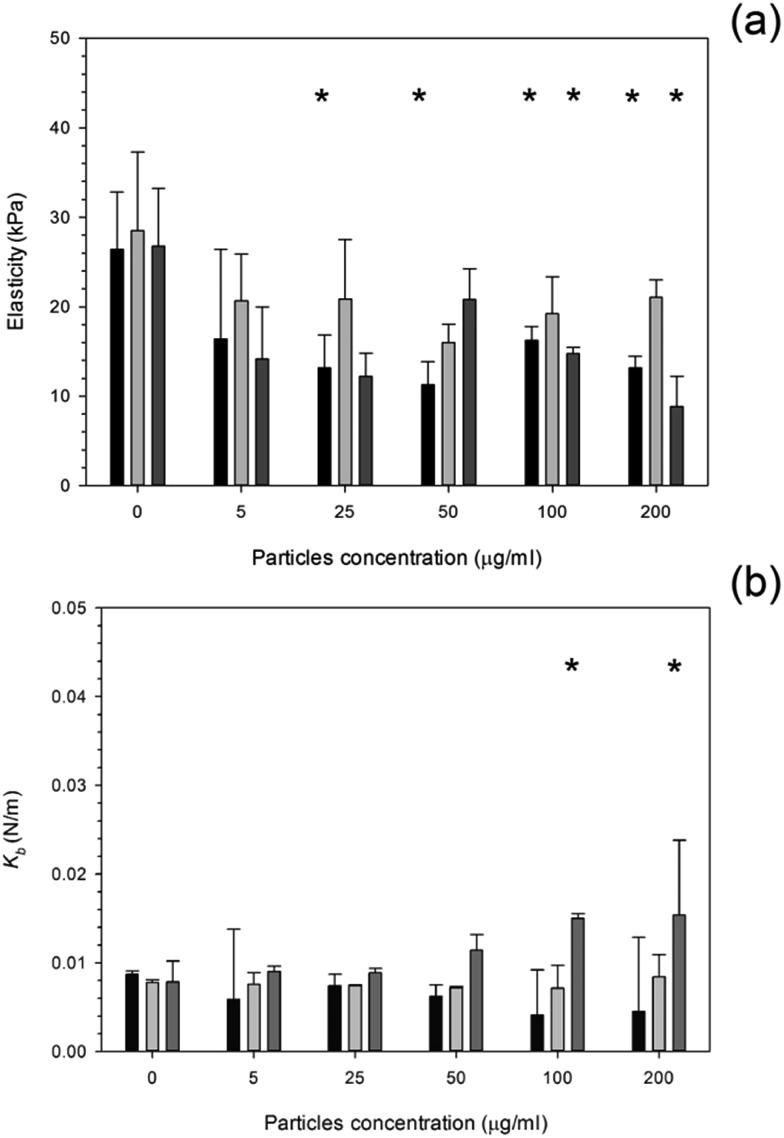
Mean cell elasticity (a), spring constant (b) of MC3T3-E1 cells exposed to PMMA particles for ( 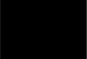 ) 24 h, ( 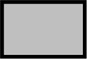 ) 48 h and ( 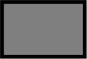 ) 72 h.

The influence of PMMA bone cement debris on osteoblast nanomechanical properties is shown in Fig. 8; after 24 hours exposure the elasticity of cell was reduced to about 14 kPa for concentrations greater than 20 μg ml^–1^; no statistical differences were determined for concentrations of PMMA debris in the range 25 to 200 μg ml^–1^ (*p* > 0.05), this was not detected after 48 hours, but with increasing exposure time (72 hours) the trend was the same as after 24 hours.

For titanium particles the spring constant values are given in Fig. 6. Similarly to the elasticity values after exposure to titanium, the spring constant values also did not increase with increasing concentration. Again the control values of spring constant were consistent over time at around 0.01 N m^–1^ (*p* > 0.05). After exposure to Ti 30 nm and Ti 100 nm, for 24 hours, the spring constant of osteoblast cells did not change with increasing concentration with a value of around 0.08 given for each concentration (*p* > 0.05); Ti 25 nm however, had the same initial value as Ti elemental at 0.08 N m^–1^ at 5 μg ml^–1^, this increased to 0.012 N m^–1^ at 12.5 μg ml^–1^, a large jump in the spring constant was observed at 25 μg ml^–1^ with a value of 0.022 N m^–1^ which slightly increased to 0.024 N m^–1^ at 50 μg ml^–1^.

After 48 hours of exposure to the titanium nanoparticles, the spring constant values were not different from the controls for all samples but the highest concentration of Ti 100 nm (*p* < 0.05).

After 72 hours of exposure, the largest titanium particles, 100 nm, had a dramatic increase in the spring constant even at the lowest concentration of 5 μg ml^–1^, no differences with nanoparticles concentration were detected (*p* > 0.05). In Fig. 7, the spring constant of osteoblast cells is shown for all time points for both cobalt nanoparticles treatments. After 24 hours Co 30 nm did not affect the spring constant for concentrations smaller than 12.5 μg ml^–1^; the spring constant then dramatically increase for the higher concentrations 25 μg ml^–1^ and 50 μg ml^–1^ at around 0.015 and 0.017 N m^–1^. For the larger particles of Co 50 nm an increase in spring constant was recorded compared to the control for all the concentrations tested (*p* < 0.05) without any difference caused by the concentration value (*p* > 0.05).

After 48 hours and 72 hours, the spring constant values increased with exposure time. No difference was recorded between types of Co nanoparticles and as result of the concentration (*p* > 0.05).

The spring constant of osteoblast cells exposed to PMMA debris is presented in Fig. 8 and revealed that only concentrations higher than 100 μg ml^–1^ for 72 hours were able to a significant increase of such parameter.

### 3.5. Metal uptake

**Fig. 9:**
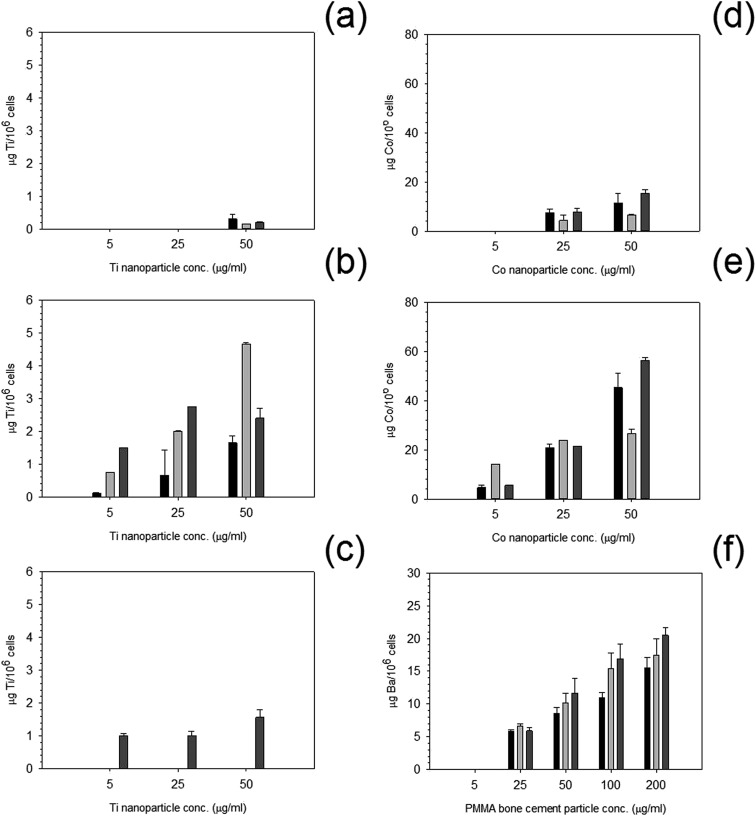
Metal uptake of MC3T3-E1 cells exposed to nanoparticles at different concentrations for 
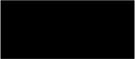
 24 h, 
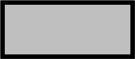
 48 h and 
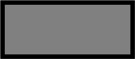
 72 h. (a) Ti 25 nm, (b) Ti 30 nm, (c) Ti 100 nm, (d) Co 30 nm, (e) Co 50 nm and (f) PMMA.

Uptake of nanoparticles increased with increasing concentration (Fig. 9); an increase in exposure time also increased the uptake of nanoparticles. Greater uptake was noted for Co 50 nm compared to Co 30 nm by almost three fold for the higher concentration of 50 μg ml^–1^ after 72 hours of exposure.

For titanium nanoparticles, overall uptake was far less than for cobalt nanoparticles, and very little to no uptake was recorded after the initial 24 and 48 hours of exposure for Ti 25 nm at the lower concentrations, with only a small uptake at 50 μg ml^–1^. For Ti 30 nm, uptake was observed at all concentrations, this increased similarly to cobalt nanoparticles with increasing concentration and increasing time. Yet, for Ti 100 nm, only after 72 hours was any uptake recorded but still followed the same pattern as other particles *i.e.* increased uptake with increasing concentration.

PMMA uptake was measured using concentration of barium, as barium is the only metal in the PMMA bone cement composition. No uptake was recorded at any time point for the lowest concentration of 5 μg ml^–1^. Although, uptake increased with increasing concentration with increasing time points similarly to both cobalt and titanium nanoparticles.

### 3.6. Cell adhesion forces

**Fig. 10:**
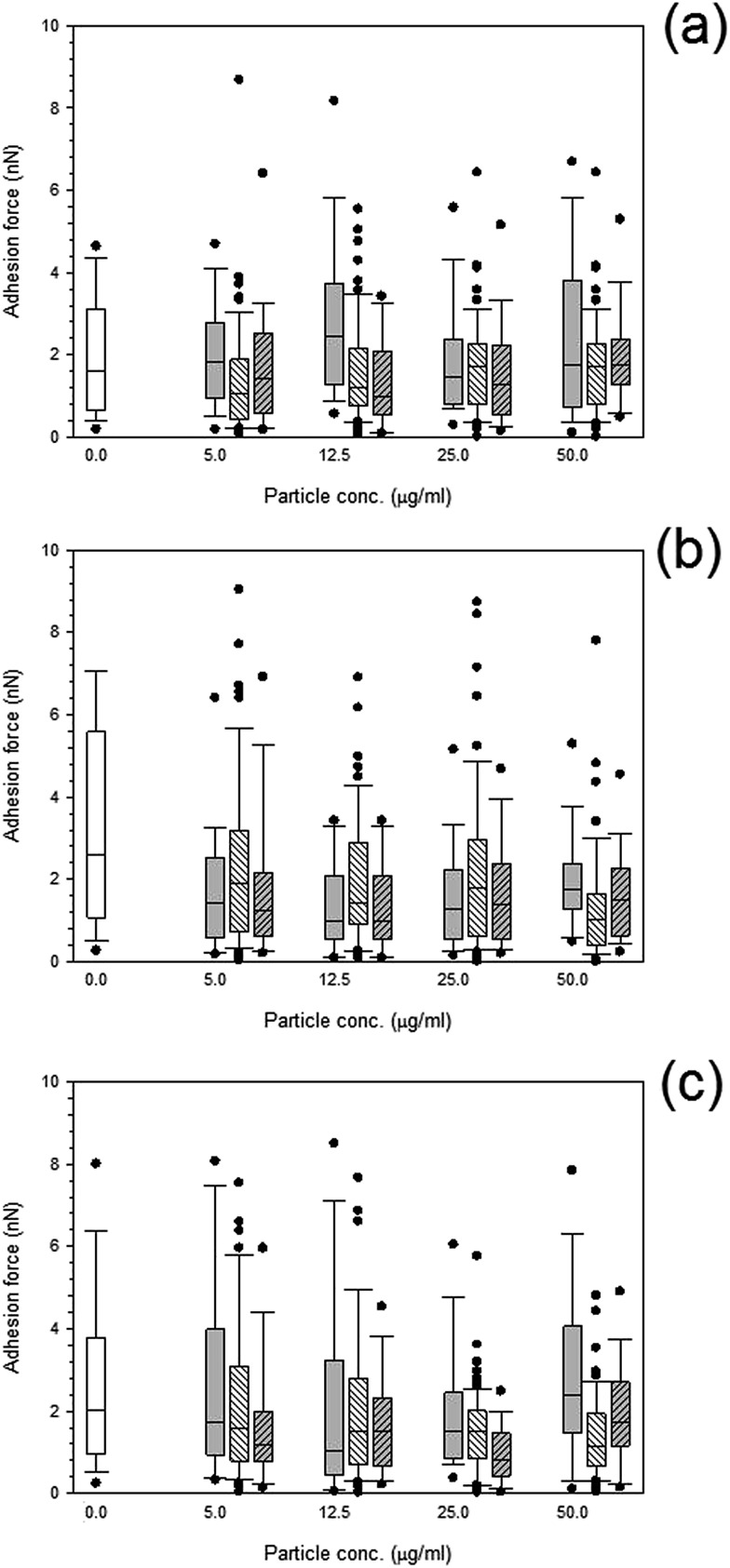
Box and whiskers plot of adhesion force distribution of MC3T3-E1 cells exposed to titanium nanoparticles for (a) 24 h, (b) 48 h and (c) 72 h. 
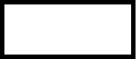
 Control, 
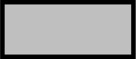
 Ti 25 nm, 
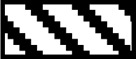
 Ti 30 nm and 
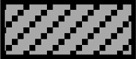
 Ti 100 nm.

**Fig. 11:**
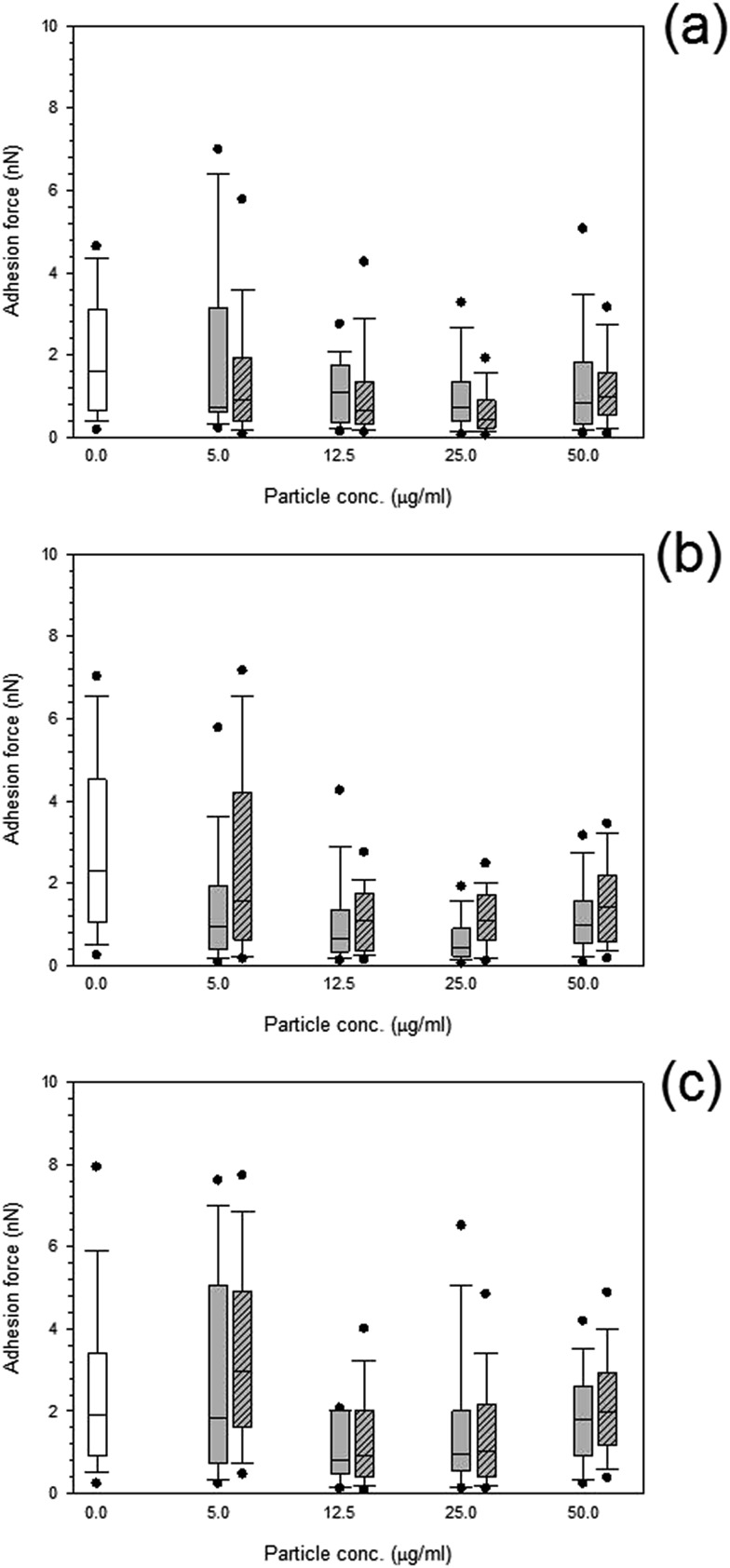
Box and whiskers plot of adhesion force distribution of MC3T3-E1 cells exposed to cobalt nanoparticles for (a) 24 h, (b) 48 h and (c) 72 h. 
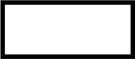
 Control, 
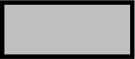
 Co 30 nm and 
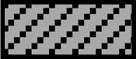
 Co 50 nm.

Both treated and non-treated cells *i.e.* not exposed to particles demonstrated spatial distributions of adhesion forces on the cell surface and were not normally distributed Fig. 10 and 11, for titanium and cobalt nanoparticles, respectively. The control sample median did not vary with increasing time with a consistent value of around 2 nN (*p* > 0.05).

Adhesion forces for cobalt nanoparticles post exposure did not differ from each other and there was no change in adhesion after exposure to either cobalt nanoparticles with increasing concentration (*p* > 0.05).

**Fig. 12:**
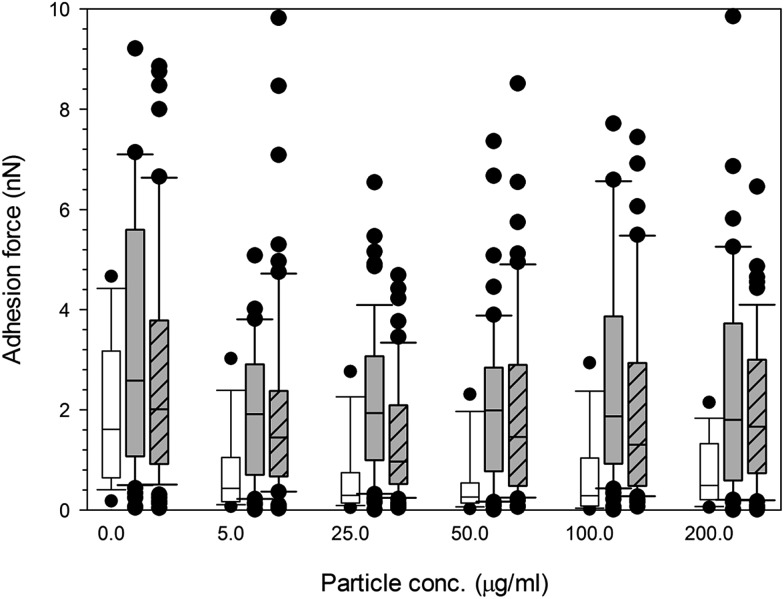
Box and whiskers plot of adhesion force distribution of MC3T3-E1 cells exposed to PMMA nanoparticles for ( 
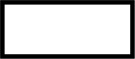
 ) 24 h, ( 
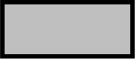
 ) 48 h and ( 
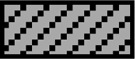
 ) 72 h.

Again, little to no difference in adhesion forces was observed for titanium after all exposure duration regardless of composition and size of the particles (*p* > 0.05) (Fig. 10). For PMMA, (Fig. 12) a general decline in cell adhesion forces were observed for cells exposed for 24 hours (*p* < 0.05). No effect of particles concentration was detected (*p* > 0.05).

## 4 Discussion

Total joint replacement (TJR) is ever increasing in a number of cases^1^ yet the leading cause of failure remains the aseptic loosening second to infection problems.^1^ Wear debris are the culprit causing aseptic loosening commonly occurring within 15 years of surgery;^1–3^ the particles produced range in size from micron to sub-micron, with the majority of wear formed less than 5 μm in diameter,^2^ and it is thought that the nano-sized range are potentially the most harmful range due to their increase in free radicals production and chromosomal damage.^13,32^ The size, shape, charge, composition and concentration range of the particles used in this study correspond to retrieved particles from explants; for example cobalt wear debris has been noted at around 50 nm, and titanium has been found in a range of sizes up to a few hundred nanometres.^33^ From abrasion, adhesion and fatigue, wear particles of PMMA bone cement were found to have a median equivalent circle diameter (ECD) *i.e.* the diameter of a circle having the same area as the measured feature, between 0.65 and 1.51 μm;^34^ however, many particles were around 10 μm, with 67% in the submicron range. In order to differentiate between the effect of both size and composition (element or oxide) of the metal nanoparticles we employed commercial nanoparticles of appropriate characteristics.

Generally, the first stages of an adverse effect is the phagocytosis of the particles by cells; this has been visually demonstrated using polystyrene-based fluorescent particles, such as fluoresbrite, that were internalised within 24 hours of *in vitro* exposure, with cells being saturated with around 40–60 particles per cell.^37^ These results are in agreement with our findings that reveal changes in the cell properties after 24 hours of exposure to nanoparticles.

Many techniques^21,38–41^ have been used to measure the mechanical properties of single cells which includes micropipette aspiration, cytoindentation, magnetic bead rheometry, optical traps and AFM.^38^ For mesenchymal stem cells, Darling *et al.* (2008),^38^ tested whether elastic and viscoelastic properties could indicate the cells phenotype, using AFM with a borosilicate functionalised tip at a spring constant of around 0.04 N m^–1^ similar to that we used in our study. Likewise, Bhadriraju and Hansen (2002)^42^ used AFM tips with spring constant of 0.06 N m^–1^ to investigate the stiffness and spreading changes of cells using AFM.^42^ In both these works, the elastic modulus was modelled using Hertz model of contact,^38,42^ as employed for our investigation. Interestingly, Darling *et al.* (2008)^38^ also found the elastic data to have a not normal distribution and that the osteoblasts demonstrated the largest elastic moduli and no difference in lineage was noted from the viscoelastic properties.

Cell elasticity is closely linked to the cytoskeleton structure,^39^ and in this study it was observed that titanium resulted in an increase in elasticity suggesting interactions with the network of stress fibres as explained by Thoumine *et al.* (1999),^39^ and disruptions to the membrane integrins of cells, forming complex disturbances to the original form which contributes to the cells rigidity.^39,44^ This could explain the changes observed in the elasticity modulus of the osteoblast cells, after PMMA particles exposure; there was a general increase in the elastic modulus with time demonstrating that cells became stiffer. It is explained^45^ that disruptions to the cytoskeleton organisation impacts on the expression of transcription factors and osteoblast-specific genes in osteoblasts,^45^ as well as influencing cell behaviour. Similar values in stiffness were observed^42^ when changes in the actin and myosin activity were investigated in relation to the changes in shape of the cell at around 20 kPa.^42^ The changes in stiffness are due to the adaptation of the cells to the stress stimuli introduced by the environment when the cells are exposed to the metal nanoparticles.^46^ Cells respond to a number of stress factors such as fluid shear stress, strain stimuli and vibration stress; such mechanosensing allows cells to detect adhesion of metal nanoparticles to the outer membrane of the cells.^46^ It had also been suggested that the cytoskeleton is the key aspect responsible for sensing the cellular mechanical changes.^46,47^

It is thought that the cytoskeleton of a cell, especially the filamentous actin determines the cells ability to maintain a stable shape, maintaining the function of the cell.^24^ It was proposed that the interactions between these actin-filament bundles of the osteoblasts cells within the cytoskeleton could cause a loss of adhesive function and, therefore, changes to the adhesion characteristics of the cell could potentially alter the cell's function.^24^ A further explanation for the changes in adhesion properties results from the decrease in cell cytoplasmic spreading and attachment area, stemming from the particles integrating to the actin filaments forming denser networks within the structure.^48^ The addition of titanium particles to the cell stress fibres reduces the spreading of the cell, this reduction in spreading area of the cell means decreased contact, thereby reducing the overall adhesion. These changes could explain the pathogenesis of peri-prosthetic osteolysis secondary to implant wear debris, with Kwon *et al.* (2001) showing a decrease in adhesion of around 40–60% in spreading attachment.^44^

Some of the above studies^11,24,35,36,37,40,41,43,44,48^ have demonstrated that orthopaedic wear debris affects the viability of cells, including their proliferation, differentiation, and function. Yet, many of these studies do not coincide, featuring many discrepancies such as the choice of cell, cell population, culturing conditions, not to mention the wear debris compositions variances such as the size, composition, dose and aggregation. However, the studies agree that particles and debris of less than 5 μm tend to interact with cells and undergo uptake *via* phagocytosis especially in regards to human, rat, and mouse bone cells. Even though results have been inconsistent for the viability assays, the biological effects are dose-dependent, and the majority of results demonstrate that the higher the concentration or dose the more adverse the effects. Non-toxic particles activate osteoblasts from the up-regulation of pro-inflammatory and bone-resorbing factors, with the down-regulation of bone forming variables. Interestingly, debris from alumina and polystyrene origins are less harmful in comparison to metals and polymers commonly used in orthopaedic devices.^6,49–51^ Our results generally do not indicate a concentration depending effect on cells properties or a spatial variation on the cell surface indicating that these nanoparticles – cells interactions are initiated at low nanoparticles concentration, possibly the lowest concentration employed in our study is already enough to saturate all the possible interactions or alternatively the responses are due to biological signalling involved in the cell detecting the presence of the particles.

From a mechanistic stand point, the presence of foreign particles in direct contact with cells causes an uptake of these particles. The complete mechanism for damage from this uptake is still unknown; however, once phagocytosis is observed it is unclear whether the physical contact or the uptake or the presence of these particles initiates a cellular response *via* membrane mediated association.

Our results also indicate that cells response is not completely linked to metal uptake as the concentration of ions inside the cell monotonically increased with exposure time whilst the nanomechanical changes were observed, particularly for the spring constant, already after the first 24 hours of contact.

Spring constant is linked to cell turgid pressure, therefore, its increase with exposure time found in our work could be linked to the metal uptake as a mechanism to prevent further ion accumulation.

As literature demonstrates that the bone adjacent to an implanted TJA has a substantial bone resorption surface.^52^ It has been assumed that wear debris contact with bone-forming cells located around loosening implants. If this contact causes an inhibition or suppression of the function of these bone-forming cells, for example damage to the cells wall, disruptions to the normal viability, proliferation, and differentiation, could lead to further loosening of the implant due to a decrease in bone renewal and formation.

It is has been reported that osteoblasts exposed to PMMA particles significantly increased their production of calcium,^53,54^ this was also true for our study. Moreover, both titanium elemental and cobalt particles did not increase the calcium formation with increasing concentration.

## 5 Conclusion

This study aimed at observing the influence of wear particles on bone cells through an alternative perspective (cells nanomechanical properties) to the usual biological responses previously investigated. Titanium increased the elasticity more than cobalt nanoparticles even though titanium demonstrated less cytotoxicity; the smaller nanoparticles had a greater impact on the viability of the cells as well as on the adhesion forces exhibited by the exposed cells. These mechanical changes are the result of alterations to the cytoskeleton as reported in literature. Also, the smaller nanoparticles had a higher uptake which may be due to phagocytosis for such smaller particles.

The results lend themselves to a novel idea of the importance of understanding the mechanical changes and their impact on normal cell function which have previously been underestimated. These results suggest that physical stimulus can alter the normal function of a cell through potential changes in the cytoskeleton of the cell in a similar manner to that of biological responses; therefore this study points out the importance of an holistically cell analysis *i.e.* not only from a biological stand point but also through mechanical mechanisms.
